# Supplementary material for: Evaluating the feasibility of automating dataset retrieval for biodiversity monitoring
Source: PeerJ. 2025 Jan 29;13:e18853. doi: 10.7717/peerj.18853 (PMC11786708; doi:10.7717/peerj.18853)
Supplement: Supplemental Information 1 [file peerj-13-18853-s001.docx]

| **Table S1**. **EBV data categories used in our framework and its synonyms.** | | | |
| --- | --- | --- | --- |
| **data type** | **Definition** | **keywords** | **keywords type** |
| presence only | presence data (at coordinates or sampling units) | presence only | synonym |
|  |  | occurrence | synonym |
|  |  | specimen records | synonym |
|  |  | specimen | synonym |
|  |  | collection | synonym |
|  |  |  |  |
| presence-absence | both presence and absence data, usually at sample units (available from designed sampling, inventories, etc.) | presence-absence | synonym |
|  |  | sampling plot | methods |
|  |  | relative proportion | methods |
|  |  | inventory | methods |
|  |  | occupancy | methods |
|  |  | quadrat | methods |
|  |  |  |  |
| abundance | number of individuals found per sample unit | abundance | synonym |
|  |  | capture-mark-recapture | methods |
|  |  | relative proportion | synonym |
|  |  | percent cover | methods |
|  |  | camera-trap | methods |
|  |  | quadrat | methods |
|  |  | counts | methods |
|  |  |  |  |
| density | number of individuals of a given species that occurs within a given sample unit or study area (available from designed sampling, inventories, etc.). | density | synonym |
|  |  | quadrat | methods |
|  |  |  |  |
| distribution | species occurrence probability over contiguous spatial and temporal units | distribution | synonym |
|  |  | range | synonym |
|  |  | habitat suitability | synonym |
|  |  | niche | synonym |
|  |  | species distribution model | methods |
| EBV genetic analysis | genetic analysis related to one of the genetic EBVs: Intraspecific genetic diversity (richness and heterozygosity) - the variation in DNA sequences among individuals of the same species; Genetic differentiation (number of genetic units and genetic distance) - divergence in genetic composition (identity and frequencies of alleles) among multiple populations. |  |  |
| non-EBV genetic analysis | genetic analysis not related to one of the genetic EBVs (e.g. gut microbiome) |  |  |
|  |  |  |  |
