# Supplementary material for: Evaluating the feasibility of automating dataset retrieval for biodiversity monitoring
Source: PeerJ. 2025 Jan 29;13:e18853. doi: 10.7717/peerj.18853 (PMC11786708; doi:10.7717/peerj.18853)
Supplement: Supplemental Information 2 — H: highly relevant, M: Moderate L: low, X: non-relevant [file peerj-13-18853-s002.docx]

| **Table S2. Main classifier relevance assignment in case of disagreement.** H: highly relevant, M: Moderate L: low, X: non-relevant | | | |
| --- | --- | --- | --- |
| Data type | Spatial range | Temporal range | Final relevance category |
| H | M (rep. L) | L (resp. M) | M |
| H | M (resp. X) | X (resp. M) | L |
| H or M | L (resp. X) | X (resp. L) | L |
| Other cases: the final score corresponds to the data type relevance | | | |
