# Supplementary material for: Evaluating the feasibility of automating dataset retrieval for biodiversity monitoring
Source: PeerJ. 2025 Jan 29;13:e18853. doi: 10.7717/peerj.18853 (PMC11786708; doi:10.7717/peerj.18853)
Supplement: Supplemental Information 3 [file peerj-13-18853-s003.docx]

| **Table S3. Number of results returned through Semantic Scholar website for each query** **(temporal range: 1980 - 2022).** | |
| --- | --- |
| Query | No of results |
| time series AND species AND Québec | 23,200 |
| abundance AND species AND Québec | 208,000 |
| density AND species AND Québec | 209,000 |
| sites AND species AND Québec | 336,000 |
| population AND species AND Québec | 448,000 |
| survey AND species AND Québec | 164,000 |
| sampling AND species AND Québec | 484,000 |
| collection AND species AND Québec | 109,000 |
| inventory AND species AND Québec | 28,600 |
| occurrence AND species AND Québec | 125,000 |
| species AND Québec | 3,490,000 |
