# Supplementary material for: Evaluating the feasibility of automating dataset retrieval for biodiversity monitoring
Source: PeerJ. 2025 Jan 29;13:e18853. doi: 10.7717/peerj.18853 (PMC11786708; doi:10.7717/peerj.18853)
Supplement: Supplemental Information 4 [file peerj-13-18853-s004.docx]

| Table S4. Most important features (top 15) of automatic classifiers for Main Classifier relevance by Support Vector Machine (stop-word removal and unigrams/bigrams selection). | | | |
| --- | --- | --- | --- |
| SVM | | | |
| No lemmatisation | | Lemmatisation | |
| **Feature** | **Importance** | **Feature** | **Importance** |
| tree  arctic  changes  genetic  population  climate  migratory  nest  fish  change  peatlands  method  stand  natal  boreal forests | 0.95  0.89  0.83  0.82  0.82  0.77  0.76  0.71  0.68  0.67  0.67  0.63  0.62  0.62  0.61 | change  arctic  tree  genetic  peatlands  egg  migratory  fish  specimen  nest  metal  swallow  climate  range  stand | 1.17  0.87  0.85  0.80  0.78  0.77  0.76  0.75  0.76  0.71  0.67  0.67  0.66  0.63  0.63 |
