# Supplementary material for: Evaluating the feasibility of automating dataset retrieval for biodiversity monitoring
Source: PeerJ. 2025 Jan 29;13:e18853. doi: 10.7717/peerj.18853 (PMC11786708; doi:10.7717/peerj.18853)
Supplement: Supplemental Information 5 [file peerj-13-18853-s005.docx]

| Table 5. Most important features (top 15) of automatic classifiers for Main Classifier relevance by Random Forest (stop-word removal and unigrams/bigrams selection). | | | |
| --- | --- | --- | --- |
| Random Forest | | | |
| No lemmatisation | | Lemmatisation | |
| **Feature** | **Importance** | **Feature** | **Importance** |
| climate  population  change  climate change  changes  structure  mechanism  particularly  distribution  conditions  quebec  areas  representing  western  genetic | 0.12  0.10  0.10  0.10  0.08  0.07  0.06  0.06  0.05  0.05  0.05  0.05  0.05  0.04  0.04 | change  climate  climate change  structure  16  species  area  particularly  population  hypothesis  range  impact  northern  year  migratory | 0.20  0.09  0.07  0.07  0.06  0.06  0.06  0.06  0.05  0.05  0.05  0.05  0.05  0.05  0.05 |
