# Supplementary figures and images for: Evaluating the feasibility of automating dataset retrieval for biodiversity monitoring
Source: PeerJ. 2025 Jan 29;13:e18853. doi: 10.7717/peerj.18853 (PMC11786708; doi:10.7717/peerj.18853)

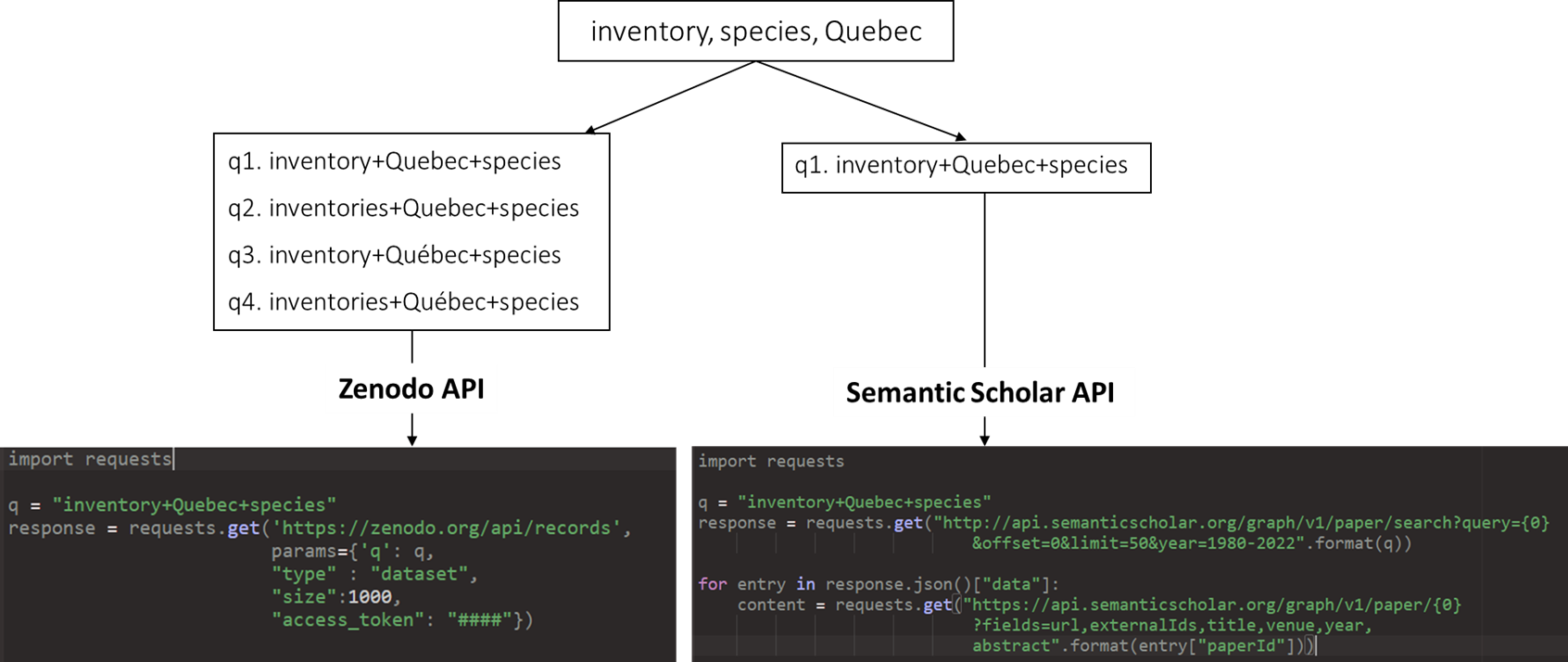

Supplement: Supplemental Information 6 — To ensure that keyword boolean combinations are properly taken into account in Zenodo’s and Semantic Scholar’s API, we first evaluated their behaviour in the management of three query formats: the accents (e.g. “Québec” or “Quebec”), the plural forms (“inventory” or “inventories”) and the order or the keywords in the boolean query. Zenodo’s API is sensitive to both plural forms and accents (i.e. the query “inventory+Quebec” does not retrieve the same results as “inventories+Quebec” nor as “inventory+Québec”). We interacted with the API using the request library, including the creation of plural and accent variations for Zenodo, and request formatting. [file peerj-13-18853-s006.png]

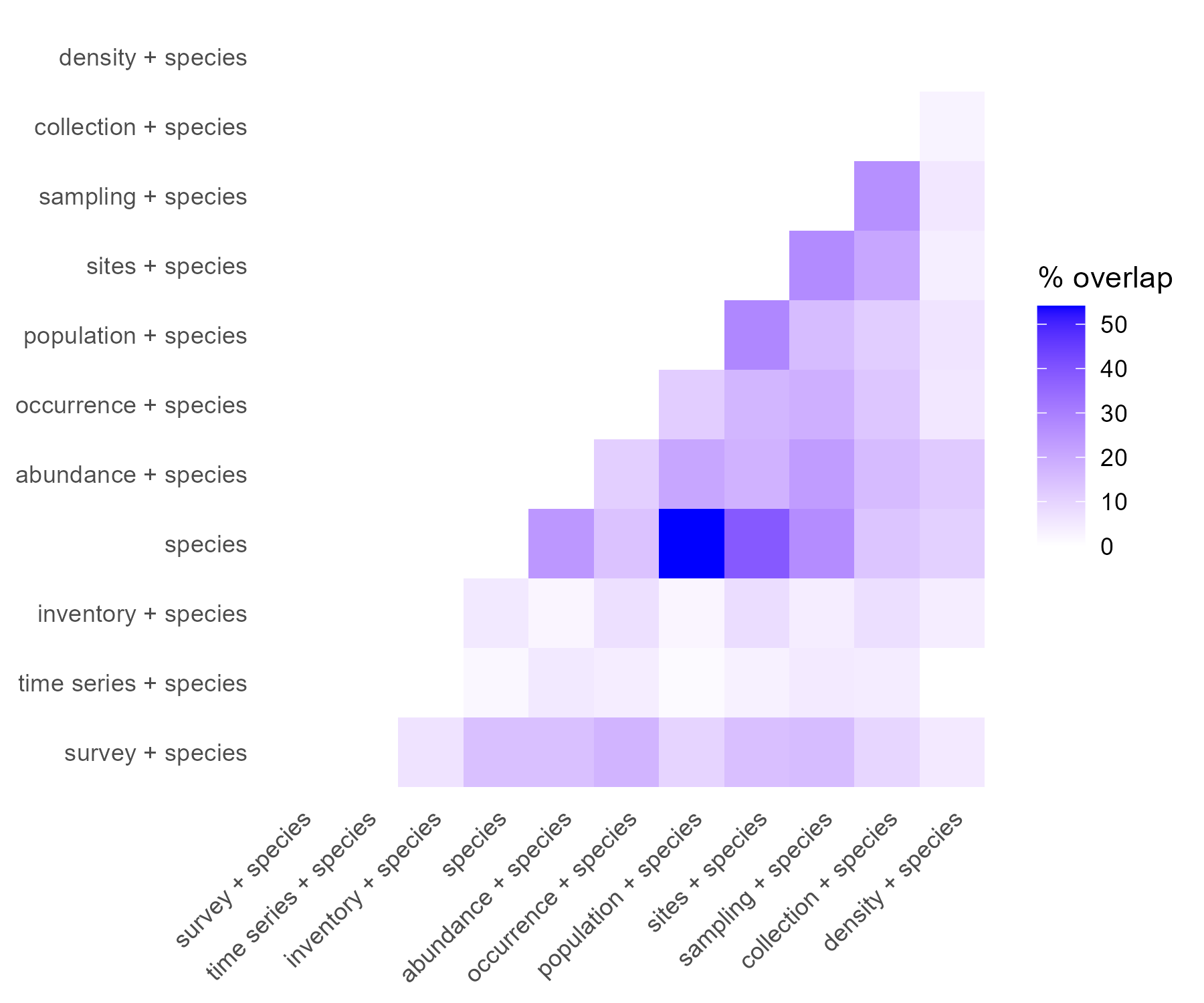

Supplement: Supplemental Information 7 [file peerj-13-18853-s007.png]
