# Supplementary material for: Evaluating the feasibility of automating dataset retrieval for biodiversity monitoring
Source: PeerJ. 2025 Jan 29;13:e18853. doi: 10.7717/peerj.18853 (PMC11786708; doi:10.7717/peerj.18853)
Supplement: Supplemental Information 8 [file peerj-13-18853-s008.docx]

**S1. Supplementary material about relevance classification**

**S1.1. Pre-processing steps for bag-of-words representation**

Pre-processing steps and classification models training were performed using NLTK (Bird et Loper 2004) and scikit-learn Python libraries (Pedregosa et al. 2011). The pre-processing steps included in the evaluation were the lemmatisation, the removal of stop-words and n-grams selection. Stop-words are frequently used words which generally do not hold any significant semantic value, such as ‘the’, ‘for’, etc. We filtered them using a predefined list of 39 English stop words from the NLTK library.

N-grams are contiguous sequences of *n* words or characters from a text. Unigrams are sequences of a single word (e.g. ‘ecology’), and bigrams are sequences of two consecutive words (e.g. ‘arctic ecology’). Taking into account bigrams might help capture local relationships between words. We represented the texts either with unigrams alone or with unigrams and bigrams, using the ngram_range parameter of the TfidfVectorizer() function from scikit-learn.

Lemmatisation converts each word into a base form (i.e. lemma), to ensure that all variations (e.g. singular and plural form) of a word are represented uniformly. We used the WordNetLemmatizer from the NLTK library.

The different combinations of pre-processing steps are detailed in Table 1, in addition to the number of features resulting from the pre-processing choice on the training corpus.

| Table S1.1. Text-processing name and corresponding combinations of pre-processing steps. | | | | |
| --- | --- | --- | --- | --- |
| **Label** | **Lemmatisation** | **Stop-words removal** | **n-grams** | **Number of features** |
| P_11 | No | No | unigrams | 2471 |
| P_12 | No | No | unigrams and bigrams | 5152 |
| P_SW_11 | No | Yes | unigrams | 2282 |
| P_SW_12 | No | Yes | unigrams and bigrams | 2907 |
| P_lem_11 | Yes | No | unigrams | 2304 |
| P_lem_12 | Yes | No | unigrams and bigrams | 5061 |
| P_lem_SW_11 | Yes | Yes | unigrams | 2116 |
| P_lem_SW_12 | Yes | Yes | unigrams and bigrams | 2852 |

**S1.2. Classifiers applied on bag-of-words representation**

We compared three classifiers: Logistic Regression (Ifrim, Bakir, et Weikum 2008), Random Forest and a linear support vector machine classifier (SVM) (Joachims 1998). For all classifiers, we used the implementation from the scikit-learn library. The three classifiers allow to take into account the class imbalance during training. We set the class_weight parameter to « balanced », which adjusts the class weights inversely proportional to their frequencies (i.e. classification performances of the majority class are penalized during the training), addressing the class imbalance in our data.

**S1.3. Classification performances**

**S1.3.1. Main Classifier**

| Table S1.2. Classification performances of the different classifiers on bag-of-words representations for the Main Classifier value, in terms of weighted precision (P), recall (R) and F1-score (F1). LR: Logistic Regression, RF: Random Forest, SVM: support vector machine. | | | | | | | | | |
| --- | --- | --- | --- | --- | --- | --- | --- | --- | --- |
|  | **LR** | | | **RF** | | | **SVM** | | |
|  | P | R | F1 | P | R | F1 | P | R | F1 |
| P_11 | 0.65 | 0.67 | 0.66 | 0.63 | 0.66 | 0.60 | 0.62 | 0.64 | 0.63 |
| P_12 | 0.67 | 0.68 | 0.67 | 0.69 | 0.69 | 0.63 | 0.65 | 0.67 | 0.65 |
| P_SW_11 | 0.68 | 0.69 | **0.68** | 0.60 | 0.64 | 0.58 | 0.64 | 0.66 | 0.64 |
| P_SW_12 | 0.67 | 0.68 | 0.67 | 0.65 | 0.67 | 0.62 | 0.66 | 0.67 | 0.66 |
| P_lem_11 | 0.66 | 0.67 | 0.66 | 0.61 | 0.65 | 0.58 | 0.64 | 0.65 | 0.64 |
| P_lem_12 | 0.67 | 0.68 | 0.67 | **0.72** | **0.71** | 0.67 | 0.67 | 0.68 | 0.66 |
| P_lem_SW_11 | 0.66 | 0.67 | 0.66 | 0.67 | 0.68 | 0.63 | 0.65 | 0.67 | 0.66 |
| P_lem_SW_12 | 0.67 | 0.68 | 0.67 | 0.69 | 0.69 | 0.65 | 0.65 | 0.66 | 0.65 |

**S1.3.2. Main Classifier and Modulators**

| Table S1.3. Classification performances of the different classifiers on bag-of-words representations for the Main Classifier and Modulators value, in terms of weighted precision (P), recall (R) and F1-score (F1). LR: Logistic Regression, RF: Random Forest, SVM: support vector machine. | | | | | | | | | |
| --- | --- | --- | --- | --- | --- | --- | --- | --- | --- |
|  | **LR** | | | **RF** | | | **SVM** | | |
|  | P | R | F1 | P | R | F1 | P | R | F1 |
| P_11 | 0.57 | 0.57 | 0.57 | 0.59 | 0.59 | 0.58 | 0.59 | 0.59 | 0.59 |
| P_12 | 0.56 | 0.56 | 0.56 | 0.54 | 0.55 | 0.54 | 0.57 | 0.58 | 0.58 |
| P_SW_11 | 0.57 | 0.57 | 0.57 | 0.54 | 0.54 | 0.54 | 0.54 | 0.54 | 0.54 |
| P_SW_12 | 0.54 | 0.54 | 0.54 | 0.55 | 0.56 | 0.56 | 0.53 | 0.53 | 0.53 |
| P_lem_11 | 0.60 | **0.61** | 0.60 | 0.54 | 0.55 | 0.54 | 0.58 | 0.58 | 0.58 |
| P_lem_12 | 0.58 | 0.58 | 0.58 | **0.61** | **0.61** | **0.61** | 0.57 | 0.57 | 0.57 |
| P_lem_SW_11 | 0.56 | 0.56 | 0.56 | 0.58 | 0.58 | 0.58 | 0.53 | 0.53 | 0.53 |
| P_lem_SW_12 | 0.54 | 0.54 | 0.54 | 0.55 | 0.55 | 0.55 | 0.53 | 0.53 | 0.53 |

**References**

Bird, Steven, et Edward Loper. 2004. « NLTK: The Natural Language Toolkit ». In *Proceedings of the ACL Interactive Poster and Demonstration Sessions*, 214‑17. Barcelona, Spain: Association for Computational Linguistics. https://www.aclweb.org/anthology/P04-3031.

Ifrim, Georgiana, Gökhan Bakir, et Gerhard Weikum. 2008. « Fast logistic regression for text categorization with variable-length n-grams ». In *Proceedings of the 14th ACM SIGKDD international conference on Knowledge discovery and data mining*, 354‑62. KDD ’08. New York, NY, USA: Association for Computing Machinery. https://doi.org/10.1145/1401890.1401936.

Joachims, Thorsten. 1998. « Text Categorization with Support Vector Machines: Learning with Many Relevant Features ». In *Machine Learning: ECML-98*, édité par Claire Nédellec et Céline Rouveirol, 1398:137‑42. Berlin, Heidelberg: Springer Berlin Heidelberg. https://doi.org/10.1007/BFb0026683.

Pedregosa, Fabian, Gaël Varoquaux, Alexandre Gramfort, Vincent Michel, Bertrand Thirion, Olivier Grisel, Mathieu Blondel, et al. 2011. « Scikit-learn: Machine learning in Python ». *Journal of machine learning research* 12 (Oct): 2825‑30.
